# Supplementary figures and images for: Expansion of intestinal Prevotella copri correlates with enhanced susceptibility to arthritis
Source: eLife. 2013 Nov 5;2:e01202. doi: 10.7554/eLife.01202 (PMC3816614; doi:10.7554/eLife.01202)

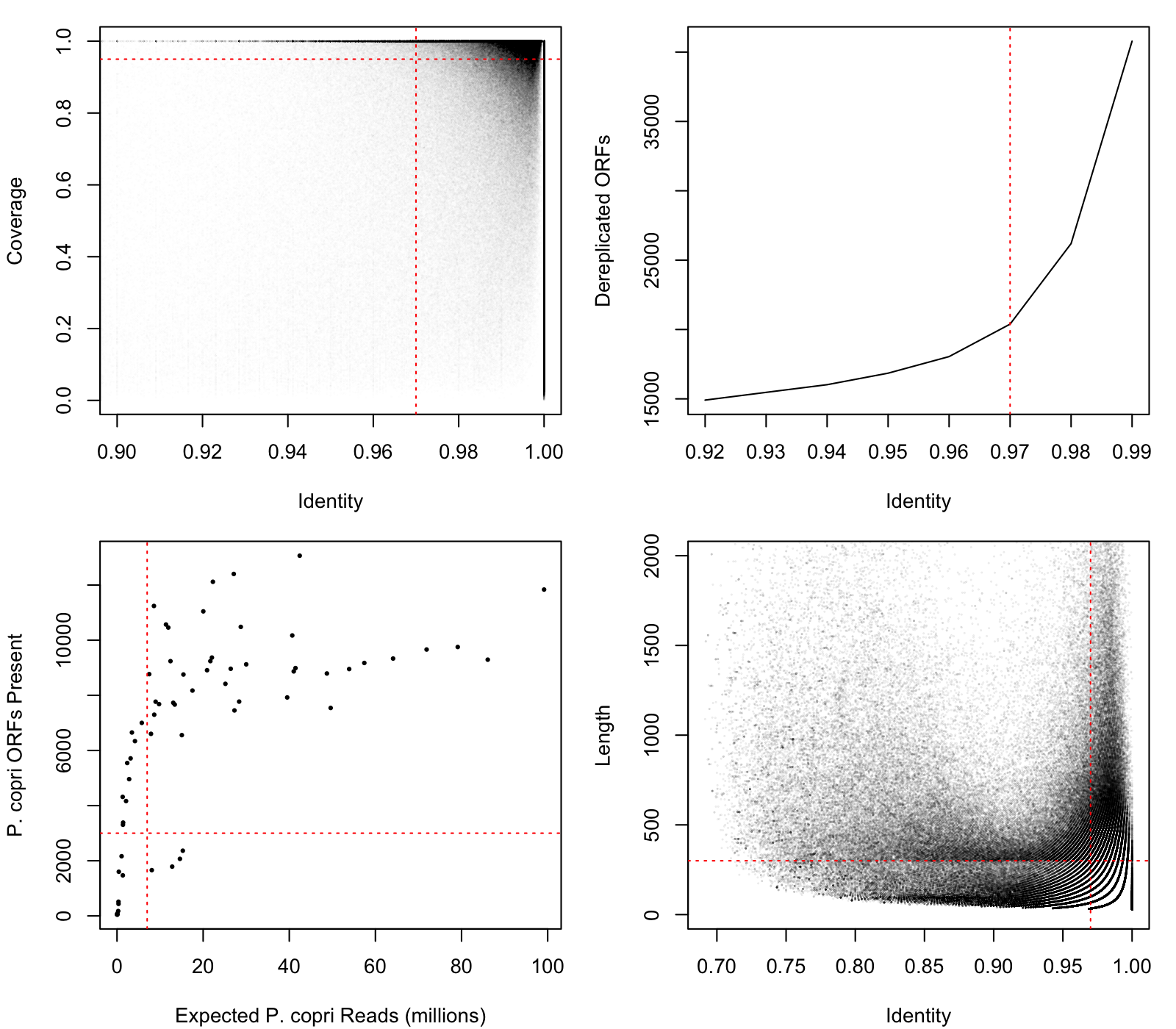

Supplement: Figure 3—source data 2. — DOI: http://dx.doi.org/10.7554/eLife.01202.016 [file elife01202s006.zip › Figure_3_supplement_1/fourpanel_figure.png]

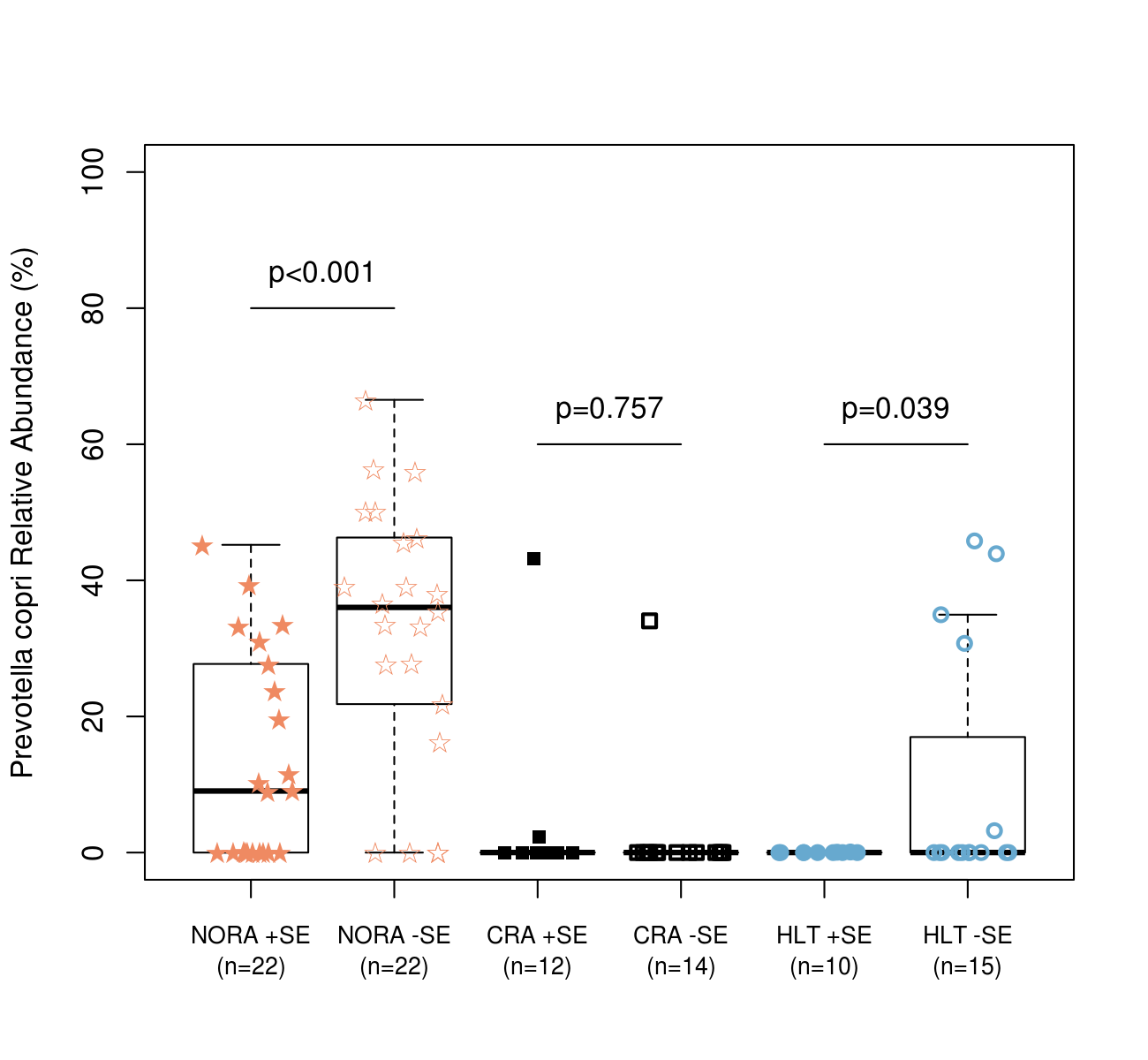

Supplement: Figure 5—source data 1. — DOI: http://dx.doi.org/10.7554/eLife.01202.022 [file elife01202s008.zip › Figure_5/figure5.png]

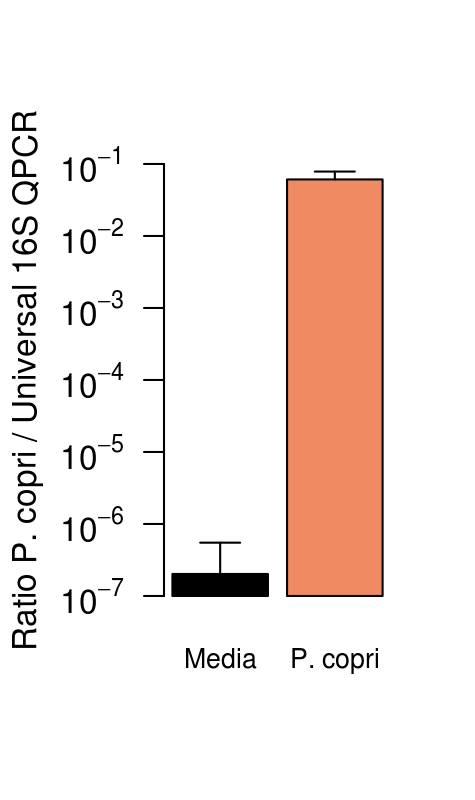

Supplement: Figure 6—source data 1. — DOI: http://dx.doi.org/10.7554/eLife.01202.024 [file elife01202s009.zip › Figure_6/panel_a.png]

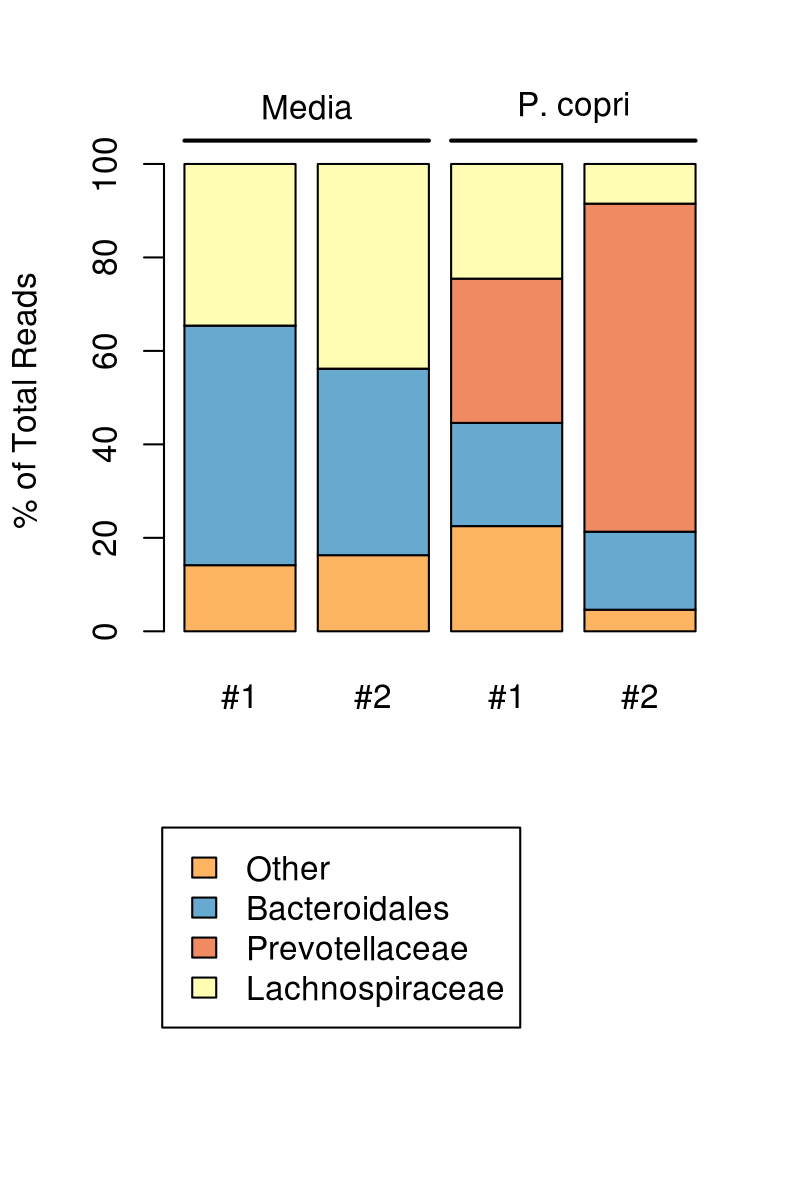

Supplement: Figure 6—source data 1. — DOI: http://dx.doi.org/10.7554/eLife.01202.024 [file elife01202s009.zip › Figure_6/panel_b-legend.png]

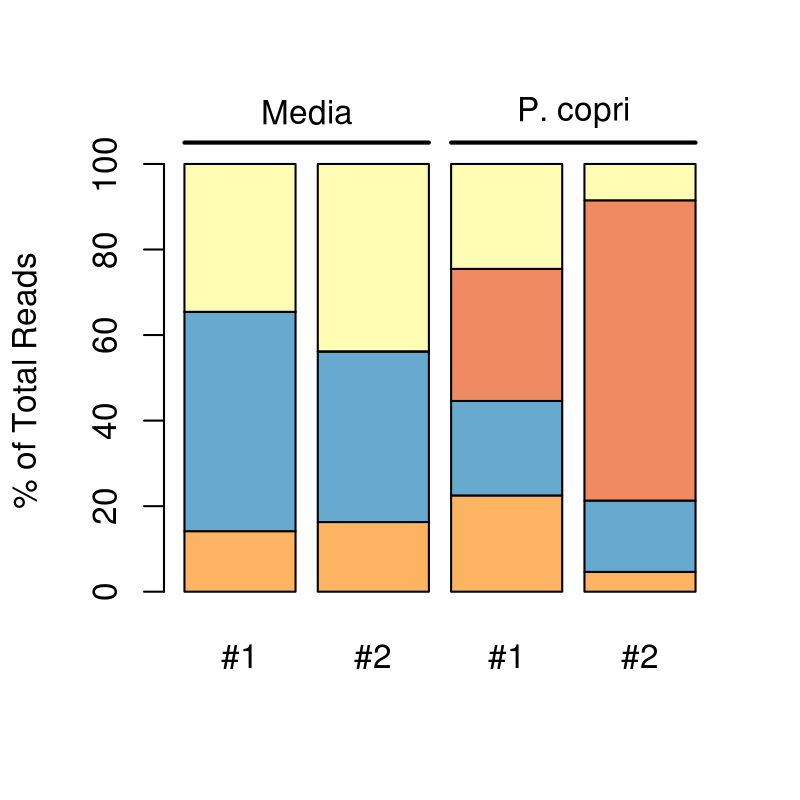

Supplement: Figure 6—source data 1. — DOI: http://dx.doi.org/10.7554/eLife.01202.024 [file elife01202s009.zip › Figure_6/panel_b.png]

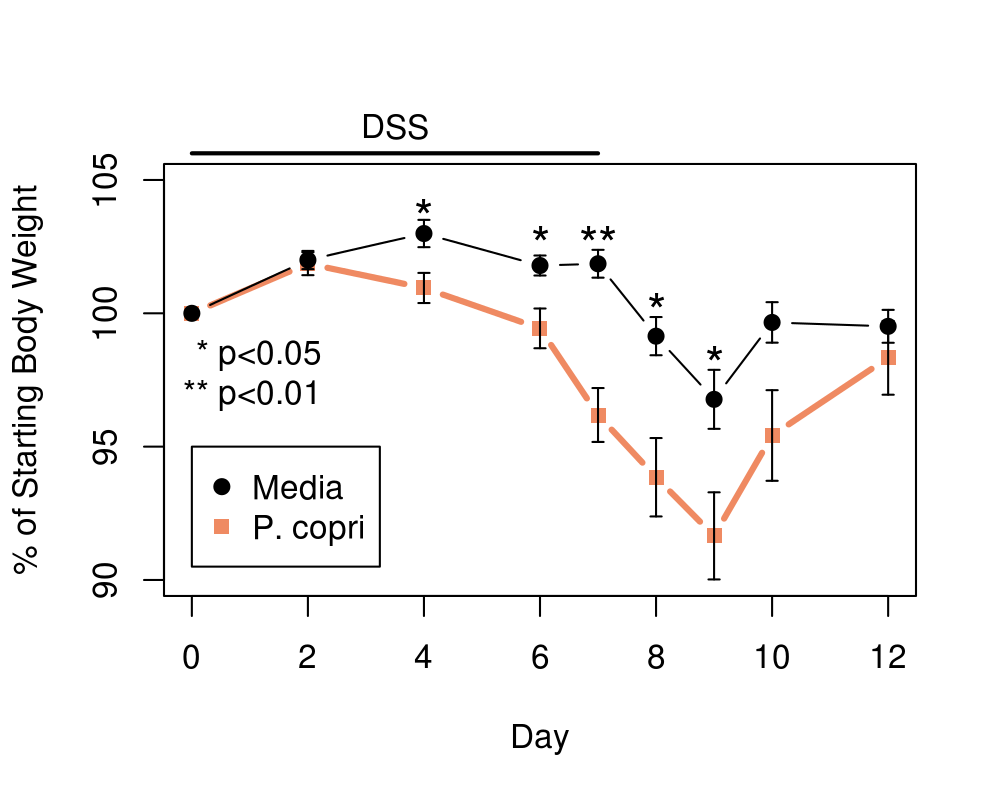

Supplement: Figure 6—source data 1. — DOI: http://dx.doi.org/10.7554/eLife.01202.024 [file elife01202s009.zip › Figure_6/panel_c.png]

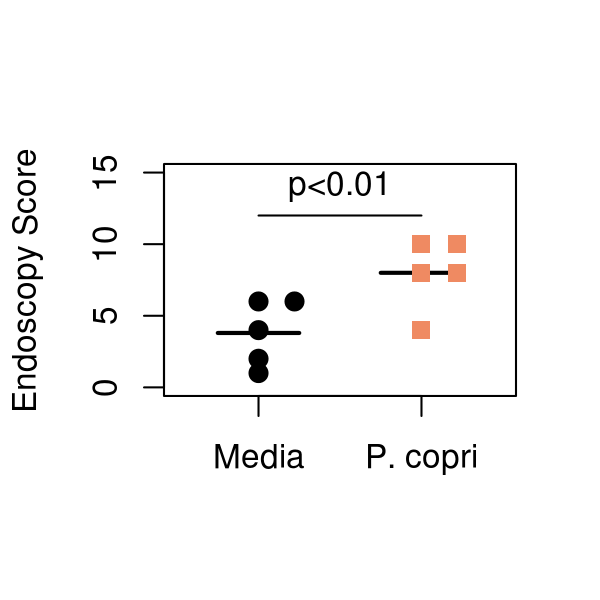

Supplement: Figure 6—source data 1. — DOI: http://dx.doi.org/10.7554/eLife.01202.024 [file elife01202s009.zip › Figure_6/panel_d.png]
